# Silica based inorganic–organic hybrid materials for the adsorptive removal of chromium

**DOI:** 10.1039/c8ra04209h

**Published:** 2018-07-02

**Authors:** Sana Nayab, Humaira Baig, Abdul Ghaffar, Eylül Tuncel, Zehra Oluz, Hatice Duran, Basit Yameen

**Affiliations:** Department of Chemistry and Chemical Engineering, SBA School of Science and Engineering, Lahore University of Management Sciences (LUMS) Lahore-54792 Pakistan basit.yameen@lums.edu.pk; Department of Chemistry, Lahore College for Women University (LCWU) Jail Road Lahore Pakistan; Department of Chemistry, University of Engineering and Technology (UET) Lahore Pakistan; Department of Materials Science & Nanotechnology Engineering, TOBB University of Economics and Technology Sögütözü Cad. 43 06560 Ankara Turkey

## Abstract

We employed polymer functionalized silica gel as an adsorbent for the removal of Cr(vi) from water. The chains of 2-aminoethyl methacrylate hydrochloride (AEMA·HCl) polymer were grown from the surface of silica gel *via* surface-initiated conventional radical polymerization and the resulting hybrid material exhibited high affinity for chromium(vi). To investigate the adsorption behavior of Cr(vi) on diverse polymer based hybrid materials, the removal capacity of (SG-AEMH) was compared with our previously reported branched polyamine functionalized mesoporous silica (MS-PEI). The adsorption capacities of polymer based materials were also compared with their respective monolayer based platforms comprising a 3-aminopropyltriethoxysilane (APTES) functionalized silica gel (SG-APTES) and mesoporous silica (MS-APTES). The polymer based systems showed excellent Cr(vi) adsorption efficiencies compared to monolayer counterparts. The structural characteristics and surface modification of these adsorbents were examined by Fourier transform infrared spectroscopy (FTIR), transmission electron microscopy (TEM), X-ray photoelectron spectroscopy (XPS), and thermogravimetric analysis (TGA). The experimental data were analyzed using the Langmuir and Freundlich models. Correlation coefficients were determined by analyzing each isotherm. The kinetic data of adsorption reactions were described by pseudo-first-order and pseudo-second-order equations. Thermodynamic parameters, *i.e.*, change in the free energy (Δ*G*°), the enthalpy (Δ*H*°), and the entropy (Δ*S*°), were also evaluated. The synthesized hybrid materials exhibited a high adsorption capacity for chromium ions. Furthermore, they could be regenerated and recycled effectively.

## Abstract 2

We employed and compared polymer functionalized silica gel and mesoporous silica as adsorbents for the removal of Cr(vi) from water.

## Graphical abstract

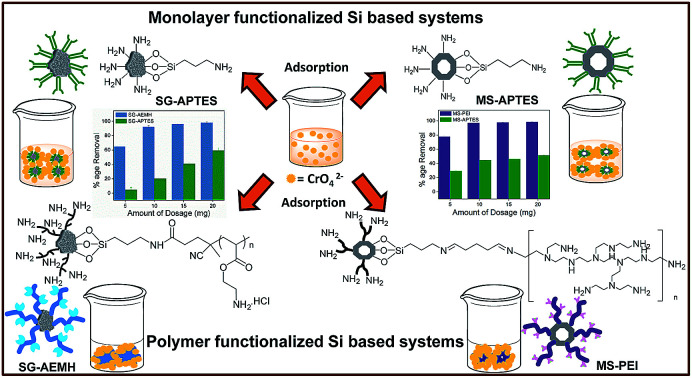

Environmental pollution has become one of the most severe problems, which is harmful to human health and ecological systems. According to recent reports, heavy metals have been considered as the most chronic and acute contaminants globally.^1,2^ Various industries such as printed board manufacturing, semiconductor manufacturing, electroplating, leather tanning, mining, steel making, textile dyes and pigments are the major sources of aquatic pollution. Industrial effluent contains different harmful heavy metals such as chromium, copper, lead, mercury.^3^ Chromium is considered highly alarming for human, animals and plants life. In wastewater, chromium exists in two stable states *i.e.*, Cr(vi) and Cr(iii). Cr(vi) is more lethal due to its solubility within almost the whole pH range and greater mobility in the waterbed.^4^ Various methods such as chemical precipitation, membrane filtration, ion exchange, electrochemical processes, chemical coagulation and adsorption have been utilized to remove heavy metals from wastewater.^5^ Among these methods, adsorption is known to be the most efficient method. A large number of natural and synthetic materials have been used for the adsorption-based removal of heavy metals from wastewater.^6–9^ These materials include zeolites, clays, biosorbents, resins, activated carbon magnetic particles and silica. Simple and low cost adsorbents have been synthesized by several researchers for an effective removal of heavy metals including Cr(vi) even at low concentration.^10–17^ Li *et al.*, demonstrated the preparation of chitosan nanofibers with an average diameter of 75 nm and cross linked with glutaraldehyde for the removal of Cr(vi).^18^ Aboutorabi *et al.*, employed TMU-30 based metal–organic framework (MOF) containing isonicotinate N-oxide as adsorptive sites for the adsorption of Cr(vi) from aqueous solution.^19^ Recently, Dong *et al.*, prepared the ionic liquid functionalized cellulose (ILFC) through the grafting of glycidyl methacrylate onto cellulose microsphere followed by reaction with ionic liquid 1-aminopropyl-3-methyl imidazolium nitrate for the adsorptive removal of Cr(vi).^20^Table 1 gives a simple comparison of the adsorption ability of different adsorbent materials for the adsorption of Cr(vi).

**Table 1:** Comparison of adsorption capacities of different adsorbents for Cr(vi) removal

Sr. no	Adsorbents	Adsorption capacity *q*_max_ (mg g^−1^)	Time (min)	pH	References
1	Carbon/boehmite (AlOOH) composite	25.6	360	2.0	51
2	Titanium oxide-Ag composite	25.7	720	2.0	52
3	Polydopamine coated maghemite NPS (MNP@PDA)	38.6	240	3.0	53
4	Fe_3_O_4_@NiO nanocomposite	6.9	40	5–10	54
5	MnFe_2_O_4_@SiO_2_-CTAB	25.0	30	3.0	55
6	ZnO/biochar	43.5	120	Natural pH	56
7	γ-AlOOH/PVA granules	35.9	200	5.5	57
8	*Yarrowia lipolytica*	5.2	120	1.0	58
9	β-Cyclodextrin ionic liquid polyurethane modified magnetic NPs (Fe_3_O_4_-CDI-IL MNPs)	2.6	180	3.0	59
10	Blends of henna with chitosan microparticles	17.4	66.21	3.8	60
11	Silver-triazolate MOF	37.0	240	6	61
12	*p*-Toluidine formaldehyde resin (PTFR) on silica	43.5	300	1.0	62
13	SG-AEMH	63.3	30	4.0	Current study
14	MS-PEI	50.26	30	4.0	Current study

Silica based porous materials are considered as promising adsorbents for water remediation due to their high surface area, well defined tunable pore size and high adsorption capacity.^21,22^ Owing to their economic feasibility, high thermal and mechanical stabilities, they can be utilized as inorganic solid matrixes in the inorganic–organic hybrid materials.^23,24^ Several researchers have contributed in the development of functionalized silica based adsorbents for the removal of heavy metals.^25–31^ Fan *et al.*, prepared the Schiff base functionalized Pb(ii) imprinted silica-supported organic–inorganic hybrid adsorbent for the selective removal of Pb(ii) from aqueous solution.^32^ Radi *et al.*, reported the synthesis of chelate β-ketoenol furan functionalized silica particles (SiNFn) for the selective adsorption of Cd(ii).^33^ More Recently, Qihui *et al.*, demonstrated the fabrication of thiol functionalized silica microspheres doped with CdTe quantum dots (CQDSMs) for the efficient adsorption of Ag^+^.^34^ The surface of silica can be tailored with different functional groups to enhance their selectivity towards specific pollutants.^35,36^ Modification can be achieved *via* post-synthesis grafting and co-condensation.^37^ Post-synthesis grafting offers a facile avenue to controlling surface properties of materials and facilitates the functionalization of the internal pores of porous materials, ultimately helping in developing material with optimized bulk and interfacial properties.^38^ Numerous organic functional groups such as amine, thiol, carboxylate, alkyl chloride, and aromatic functional groups have been incorporated through post-synthesis grafting strategy.^39–44^ In case of silica based materials, the silanol groups present on the surface assist the covalent introduction of a wide range of functional groups, which act as stable and efficient chelating moieties towards a variety of metal ions. The excellent metal adsorption property of these functionalized silica materials are attributed to the presence of electron donor heteroatoms such as O, S and N in the incorporated functional groups.^45,46^ The surface functionalization can be either monolayer or polymer based. The polymer based surface functionalization results in a higher surface functional group density that ultimately improves the absorption capacity of the functionalized material. Despite obvious advantages of the polymer based surface functionalization, majority of the efforts in the field of developing materials for water remediation have been focused on monolayer based surface functionalizations.

**Scheme 1:**
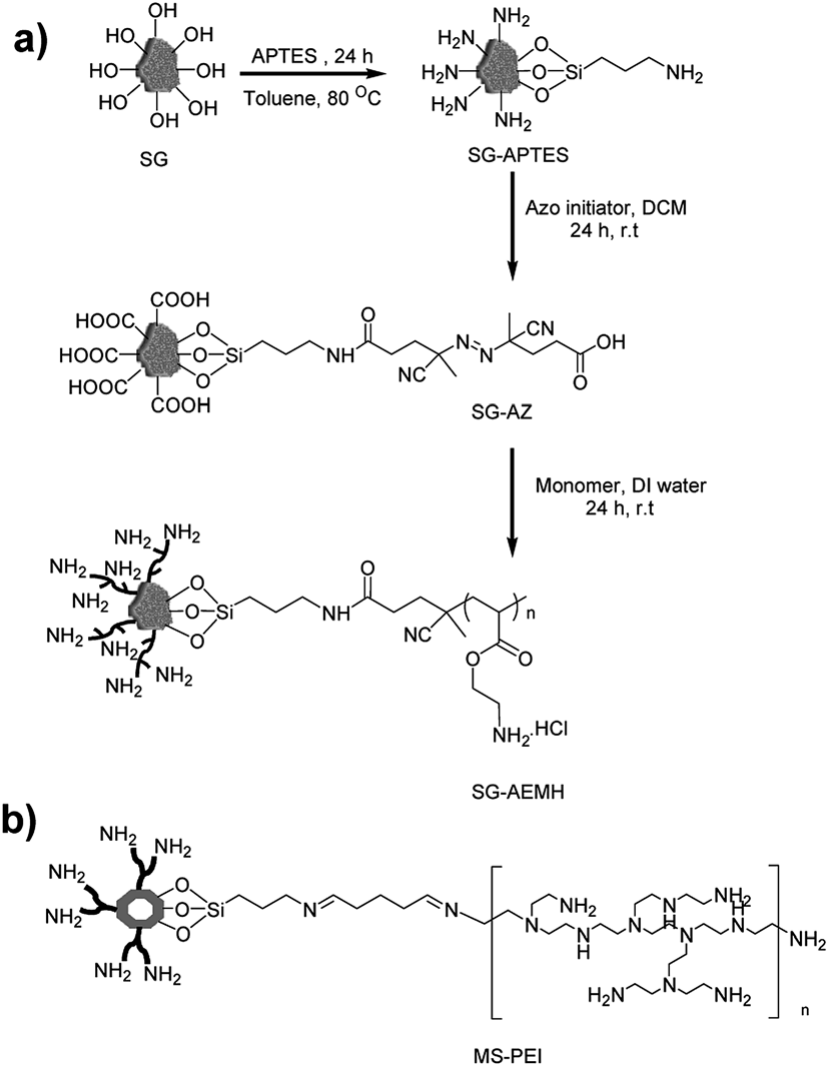
Schematic illustration of (a) synthesis of APTES based monolayer (SG-APTES) and AEMH based polymer functionalized silica gel (SG-AEMH), (b) polyamine functionalized mesoporous silica (MS-PEI).

Herein, we demonstrated the potential of polymer functionalized silica based inorganic–organic hybrid materials for Cr(vi) adsorption (Scheme 1). The chains of 2-aminoethyl methacrylate hydrochloride were grafted on the surface of silica gel *via* surface-initiated conventional radical polymerization (SI-cRP) approach. We have also compared the adsorption capacity of SG-AEMH with polyamine functionalized MCM-41 mesoporous silica (MS-PEI).^47^ The APTES derived monolayer based amine functionalized silica gel (SG-APTES) and mesoporous silica (MS-APTES) were also examined and compared with polymer grafted silica materials. Our results show that SG-AEMH and MS-PEI were more effective for chromium adsorption. Furthermore, the experimental data were fitted to different adsorption models, and the corresponding parameters were determined. In addition, kinetic and thermodynamic analyses were performed to understand the mechanism of the adsorption processes.

## Experimental

### Materials and methods

Silica gel (column chromatography grade, 0.06–0.2 mm) and potassium dichromate were purchased from Scharlau, Spain. Hydrochloric acid (37%) and toluene (99%) were purchased from Riedel-de Haën, Germany. 2-Aminoethyl methacrylate hydrochloride, Triethylamine (TEA), 1-hexadecyl trimethylammonium bromide (CTAB), aqueous ammonia (NH_3_, 35%), tetraethyl orthosilicate (TEOS, 99%), toluene (99%), 3-aminopropyltriethoxysilane (APTES, 98%), glutaraldehyde (GA, 50% in water), branched polyethyleneimine (PEI, *M*_w_ ∼25 kDa by LS, *M*_n_ ∼10 kDa by GPC, data from Sigma Aldrich), sodium dihydrogen phosphate (NaH_2_PO_4_, 97%), sodium hydrogen phosphate (Na_2_HPO_4_, 98%), ethanol (>99%), acetone and methanol were purchased from Sigma Aldrich, Germany. Acetic acid was obtained from Merck, Germany. TEA was refluxed overnight with calcium hydride, distilled, and stored under a nitrogen atmosphere. 4,4′-Azobis(4-cyanopentanoyl chloride) (ACPC) was synthesized from 4,4′-azobis (4-cyanopentanoic acid) according to a previously reported method.^48^ Toluene was dried using Na/benzophenone prior to use.

### Activation of silica gel surfaces: (SG)

Silica gel was activated by stirring its suspension in conc. HCl for 24 h at ambient temperature. The acid suspension was subsequently diluted with deionized water and activated silica gel was separated by centrifugation (4000 rpm, 10 min). The activated silica gel was washed with deionized water until neutral and dried under vacuum at 90 °C for overnight.

### Synthesis of APTES functionalized silica gel (SG-APTES)

Amine functionalized silica NPs were prepared by a previously reported method.^47^ Activated silica gel (4 g) and 10% APTES solution (60 mL) were added in dry toluene and refluxed at 80 °C for 24 h under inert atmosphere. The reaction mixture was cooled and silica gel was separated by centrifugation at 4000 rpm for 10 min followed by washing with toluene, acetone and methanol. The APTES functionalized silica gel was dried in a vacuum oven at 70 °C for overnight.

### Surface modification of silica gel with azoinitiator (SG-AZ)

The surface of silica gel was further modified with azoinitiator according to the previously reported method.^49^ A solution of ACPC (4,4-azobis 4-cyanopentanoylchloride) (0.5 g) was prepared in 17 mL of dry dichloromethane, followed by the addition of dry TEA (216 µL) under inert atmosphere. This solution was injected over degassed APTES functionalized silica gel (SG-APTES 2 g) under nitrogen flow and stirred for 2.5 h at ambient temperature. The particles were separated by centrifugation (4000 rpm), followed by washing with DCM and ethanol. The particles were stored in refrigerator until further use.

### Grafting of poly AEMH·HCl brushes on the surface of silica gel (SG-AEMH)

AEMH·HCl monomer (2.7 g) was dissolved in 13 mL deionized water and solution was degassed for 1 h at room temperature. The monomer solution was transferred to a Schlenk containing already degassed azoinitiator coated silica gel (0.4 g). The polymerization was carried out under N_2_ (gas) at 75 °C for 24 h. Polymer functionalized silica gel was separated by centrifugation (4000 rpm), washed with water and dried in a vacuum oven at ambient temperature for 24 h.

## Characterization

Attenuated total reflection Fourier transform infrared (ATR-FTIR) spectra were recorded on Alpha Bruker, spectrometer (Germany). Transmission electron microscopic (TEM) images were obtained on FEI Tecnai G2 F20 instrument with an accelerating voltage of 200 kV. Samples were prepared by drop casting two to three drops of particle dispersions in ethanol onto a carbon coated copper TEM grid. X-ray photoelectron spectroscopy (XPS) measurements were carried out using Thermo Scientific K-Alpha. The Mg Kα (1253.6 eV) X-ray source was operated at 300 W. Pass energy of 117.40 eV was used for the survey scans. The spectra were recorded using a 60° take off angle relative to the surface normal. The UV/Vis absorption spectra were recorded using a Shimadzu UV-1800 spectrophotometer. Thermogravimetric measurements were carried out on a TGA Q50 V6.2 Build 187 thermogravimetric analyzer. Samples were heated at 10 °C min^−1^ from ambient temperature to 800 °C under nitrogen flow.

## Adsorption studies

The adsorption studies were carried out by investigating the effect of different pH. The pH values were adjusted by using 0.1 M HCl and 0.1 M NaOH. Approximately, 10 mg of adsorbents were shaken at room temperature (200 rpm) with 10 mL aqueous Cr(iv) solutions of known initial concentration (40 ppm for SG-APTES and SG-AEMH, while 20 ppm for MS-APTES and MS-PEI) at optimized contact time. At the end of the adsorption period, the solutions were centrifuged and the concentration of Cr(iv) in the supernatant solutions before and after the adsorption was determined using a calibration curve (*λ*_max_ 353 nm). The amount of metal adsorbed at equilibrium *q*_e_ (mg g^−1^) was calculated from the following equation.1
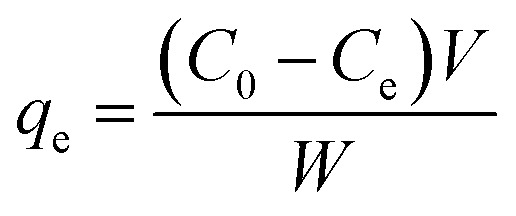
where *q*_e_ is the adsorption capacity (mg g^−1^) of the adsorbent at equilibrium, *C*_0_ and *C*_e_ (mg g^−1^) are the initial and equilibrium concentrations of solute, *V* is the volume of the aqueous solution in liter, and *W* is the mass in grams of the adsorbent used.

## Result and discussion

**Fig. 1:**
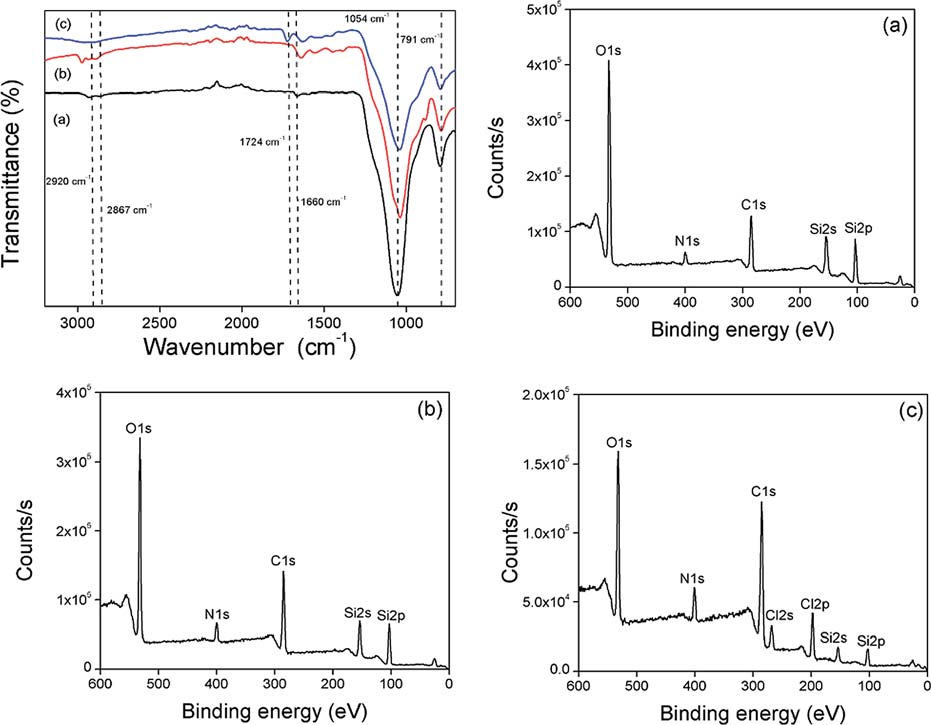
FTIR spectra of (a) SG-APTES, (b) SG-AZ, (c) SG-AEMH; XPS spectra of (a) SG-APTES, (b) SG-AZ, and (c) SG-AEMH.

**Fig. 2:**
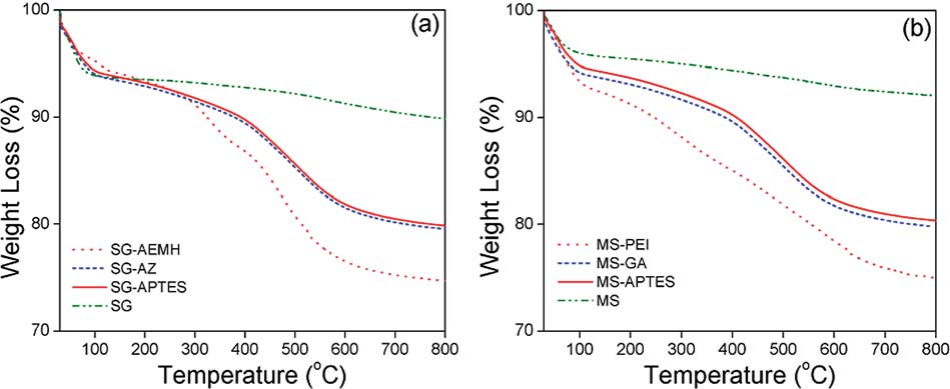
TGA analysis of (a) as synthesized SG, SG-APTES, SG-AZ, SG-AEMH (b) as synthesized MS, MS-APTES, MS-GA, MS-PEI.

The surface functionalization of silica gel with monolayer and polymer was affirmed by FTIR spectroscopic analysis (Fig. 1). The bands at 1054 cm^−1^ and 791 cm^−1^ are characteristic of asymmetric and symmetric vibrations of Si–O–Si. The surface modification of SG with APTES was confirmed by the appearance of –NH_3_^+^ bending vibration at 1583 cm^−1^ followed by the presence of NH_2_ bending vibration at 1660 cm^−1^ and C–H (CH_2_) stretching vibration at 2867 cm^−1^ and 2920 cm^−1^. The C=O stretching vibration at 1724 cm^−1^ and N–H stretching vibration of at 3330 cm^−1^ further supported the immobilization of AEMH on the surface of silica gel. The successful surface modifications were further established by XPS analysis (Fig. 1). The survey scan of SG-APTES showed signals at 143 and 100 eV, which correspond to the binding energies of Si 2s and Si 2p orbitals of silicon. The signal for the C 1s and O 1s orbitals of the carbon and oxygen contents can be observed at 283 and 532 eV. The presence of N 1s orbital signal at 400 eV in the XPS survey scan supported the amine functionalization of SG. In case of SG-AEMA, the XPS survey scan also showed the signal for Cl 2s (268 eV) and Cl 2p (198 eV), because the monomer used for the polymer brush growth was in its hydrochloride form. Thermogravimetric analysis was conducted to evaluate the extent of surface functionalization (Fig. 2). The pristine SG and MS exhibited a total weight loss of 9.92% and 8.38% respectively at temperatures up to 800 °C, which was attributed to the weight loss by the removal of silanol groups. In the case of SG-APTES and MS-APTES, the weight loss was 19.55% and 20.87%, respectively, which was attributed to the decomposition of monolayer of APTES. By grafting the polymer onto the surface of the silica materials, the weight loss rises sharply to 25.90% for SG-AEMH and 24.32% for MS-PEI.

**Fig. 3:**
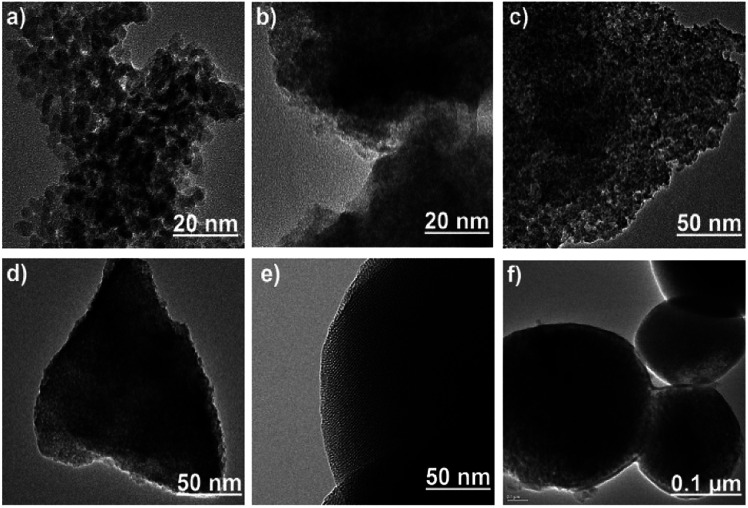
The HR-TEM images of (a) SG (b) SG-APTES, (c) SG-AZ, (d) SG-AEMH, (e) MS-APTES, and (f) MS-PEI.

In case of silica gel, the evaluation of surface functionalization by TEM (Fig. 3a and f) was limited by the large variation in size and relatively thin layer of the surface-immobilized monolayer and polymer. SG forms large clusters size ranges from few micrometres to few hundred nanometres. MS samples, on the other hand, have more regular shapes with narrower size distribution (∼500 nm). The mesopores of MS were also evident in the TEM images. The TEM images of MS-PEI revealed a thin layer of PEI coated on the surface of MS.

### Effect of pH

**Fig. 4:**
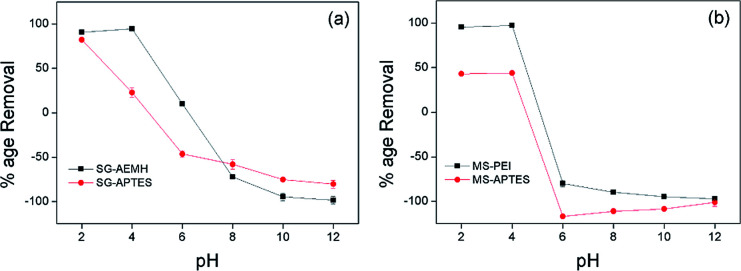
Effect of pH on the adsorption of Cr(vi) by (a) SG-AEMH, SG-APTES, (b) MS-PEI, MS-APTES.

The pH value of the medium controls the adsorption capacity due to its influence on the ionic forms of the chromium ions in solutions, surface change and protonation degree of functional groups on the adsorbent. Cr(vi) exists in five main forms in the aqueous solution, including Cr_2_O_7_^2−^, HCr_2_O_7_^−^, CrO_4_^2−^, HCrO_4_^−^ and H_2_CrO_4_. CrO_4_^2−^ is dominant at pH > 6.0, while HCrO_4_^−^ and Cr_2_O_7_^2−^ exist in equilibrium between pH 2 and pH 6. Below pH 1, Cr(vi) species are present as H_2_CrO_4_ and HCr_2_O_7_^−^. To evaluate the adsorption of Cr(vi) onto the developed adsorbents (SG-APTES, SG-AEMH, MS-APTES, and MS-PEI) in the pH range of 2.0–12.0, 10 mg of adsorbents were added in 10 mL Cr(vi) solution (40 ppm for SG-APTES and SG-AEMH, while 20 ppm for MS-APTES and MS-PEI) and placed in a shaker (180 rpm) at room temperature for 30 min. The pH of solutions was adjusted by using 0.1 M HCl and 0.1 M NaOH. The adsorption capacity of all the adsorbents increased as the pH increased from 2.0 to 4.0 and then decreased as the pH increased from 4.0 to 12.0 for all the adsorbents. The maximum adsorption capacities of polymer functionalized silica materials were observed at pH 4.0 (93% for SG-AEMH and 98% for MS-PEI). The increase in the adsorption capacity at low pH might be attributed to the conversion of Cr(vi) species into HCrO_4_^−^ and Cr_2_O_7_^2−^ and increase in the extent of protonation of the amino groups. The decrease in the adsorption capacity at higher pH was attributed to the decrease in the extent of protonation of the amino groups on the silica gel and strong competition between OH^−^ and CrO_4_^2−^ ions. This indicated that the electrostatic interaction and ion exchange played important roles in the adsorption of Cr(vi) (Fig. 4).^50^

### Effect of the amount of adsorbent

**Fig. 5:**
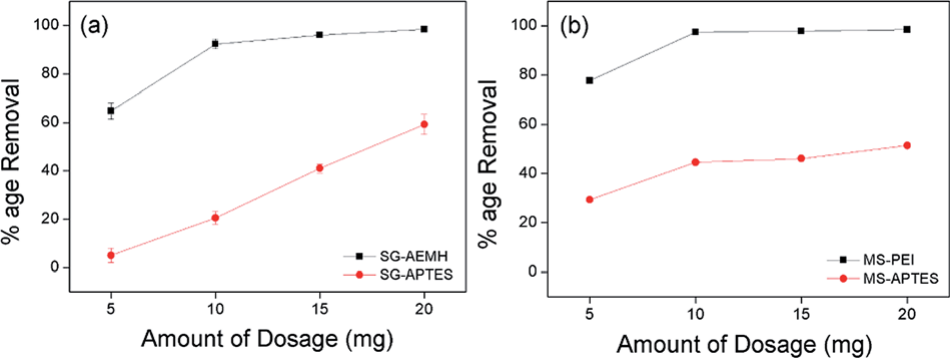
Effect of amount of dosage on the adsorption of Cr(vi) by (a) SG-AEMH, SG-APTES, (b) MS-PEI, MS-APTES.

The amount of adsorbent is an important key in the process of adsorption. The effect of the dosage amount was investigated by adding different amounts of adsorbents (5 10, 15, and 20 mg) in 10 mL of Cr(vi) solution (40 ppm for SG-APTES and SG-AEMH, while 20 ppm for MS-APTES and MS-PEI) at pH 4. Increase in Cr(vi) adsorption capacity was observed by increasing the amount of adsorbents. SG-AEMH and MS-PEI showed higher adsorption than SG-APTES and MS-APTES (Fig. 5).

Maximum adsorption was achieved at 20 mg for SG-AEMH (98%) and at 10 mg for MS-PEI (98%). This could be attributed to the increase in the adsorbent specific surface area and availability of more adsorption sites.^63^

### Effect of contact time

**Fig. 6:**
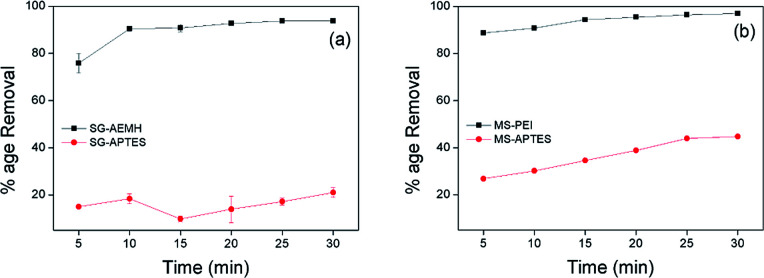
Effect of contact time on the adsorption of Cr(vi) by (a) SG-AEMH, SG-APTES, (b) MS-PEI, MS-APTES.

Adsorbent needs to show rapid uptake of pollutants for an ideal and practical adsorption process. To investigate the adsorption capacity of silica sorbents as a function of time, different adsorbents (10 mg) developed in this study were added in 10 mL of Cr(vi) solution (40 ppm for SG-APTES and SG-AEMH, while 20 ppm for MS-APTES and MS-PEI) separately and percentage removal was monitored at room temperature at 5 minutes time intervals for 30 minutes. The uptake of adsorbate increased with contact time. SG-AEMH and MS-PEI showed higher adsorption capacities at any time slot than SG-APTES and MS-APTES. All adsorbents under study exhibited maximum adsorption after 30 min and thereafter no significant change in removal was observed. Adsorption was 93% for SG-AEMH and 21% for SG-APTES while 98% adsorption was achieved for MS-PEI and 44% for MS-APTES. The rapid adsorption performance of adsorbents might be related to the availability of greater number of active sites in beginning but as the time increases, active surfaces become saturated with adsorbate species. It was rational to assume that the fast adsorption equilibrium was not only due to strong chelation and good affinity of the sorbents towards Cr(vi) (Fig. 6).^64^

### Effect of initial Cr(vi) concentration

**Fig. 7:**
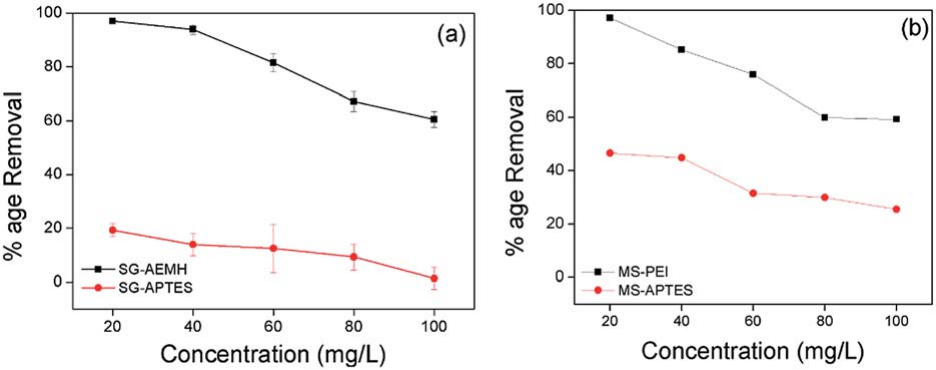
Effect of initial concentration of Cr(vi) on the adsorption by (a) SG-AEMH, SG-APTES, (b) MS-PEI, MS-APTES.

To investigate the effect of initial concentration on the metal removal capability of adsorbents, adsorption was carried out at different initial concentrations (20, 40, 60, 80, 100 mg L^−1^) with 10 mg of adsorbents. It was observed for all the adsorbents that an increase in Cr(vi) concentration resulted in the decrease in Cr(vi) removal capacity (Fig. 7). This trend may be attributed to the lesser number of available active sites for the adsorption against increased Cr(vi) concentration.^65^

**Fig. 8:**
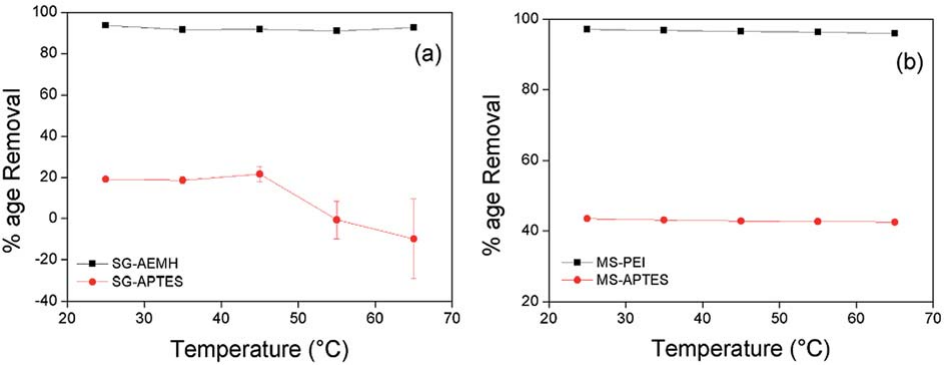
Effect of temperature on the adsorption of Cr(vi) by (a) SG-AEMH, SG-APTES, (b) MS-PEI, MS-APTES.

### Effect of temperature

Temperature plays an important role in the process of adsorption. To study the effect of temperature on the adsorption capacity, adsorption was performed at different temperatures (25, 35, 45, 65 °C). 10 mg of adsorbents were added into 10 mL chromium metal solution (40 ppm for SG-APTES and SG-AEMH, while 20 ppm for MS-APTES and MS-PEI) and stirred for 30 min at pH 4. For all the adsorbents, the adsorption capacity decreased with an increase in temperature. This was attributed to the fact that with increase in temperature the interaction between the metal ions and adsorbents became weak. The highest percentage removal (93% for SG-AEMH and 98% for MS-PEI while 21% for SG-APTES and 44% for MS-APTES) was observed at room temperature (Fig. 8).^66^

### Adsorption isotherms

The adsorption isotherm facilitates in understanding the relationship between the adsorbate and adsorbent. Langmuir,^67^ Freundlich^68^ isotherms were employed to express the adsorption data. The Langmuir isotherm assumes the monolayer adsorption of metal ions on the homogeneous adsorbent surface with a finite number of adsorption sites and is expressed by the following equation.2
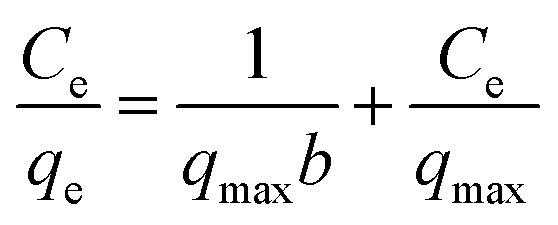
where *q*_e_ is the amount of adsorbed metals ions in the sorbent (mg g^−1^), *C*_e_ is the equilibrium metal ion concentration in solution (mg L^−1^), *b* (L mg^−1^) is the equilibrium constant related to the adsorption energy, and *q*_max_ is the maximum adsorption capacity (mg g^−1^). In addition, the viability of adsorption of Cr(vi) can be expressed by using a dimensionless factor, called separation factor (*R*_L_), which may be defined by following equation:3
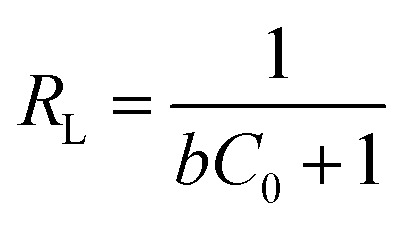
where *b* is the Langmuir constant (L mg^−1^) and *C*_0_ refers to the initial metal ions concentration (mg L^−1^). The value of *R*_L_ related to the shape of isotherm indicates whether the adsorption is irreversible (*R*_L_ = 0), linear (*R*_L_ = 1) favourable (0 < *R*_L_ <1) or unfavourable (*R*_L_ > 1).

The Freundlich isotherm is based on the assumption that the adsorbate adsorbs onto the heterogeneous adsorbent surface and is not restricted to monolayer formation. The linear form of the Freundlich isotherm is represented by the following equation:4
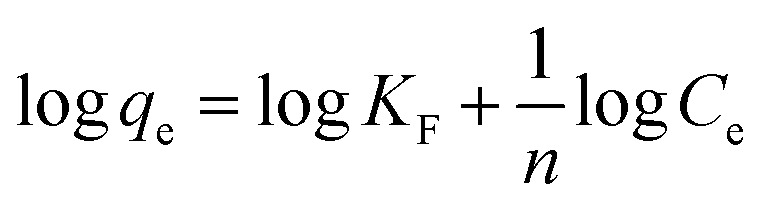
where *K*_F_ is the Freundlich isotherm constant related to adsorption capacity. *C*_e_ and *q*_e_ are the equilibrium concentration of adsorbate in solution and on adsorbent respectively. The slope 1/*n* (with favorable range between 0 and 1) is the measure of surface heterogeneity and adsorption intensity, respectively. The lower the value of 1/*n*, the more heterogeneous is the adsorption process. Table 1 summarizes both the Langmuir and the Freundlich parameters, together with the correlation coefficients.

Table 2 summarizes both the Langmuir and the Freundlich parameters, together with the correlation coefficients. It can be observed that for SG-AEMH the Langmuir model provided a good fit to the experimental data with high *R*^2^ (0.99) value compared to the Freundlich model *R*^2^ (0.87)

**Table 2:** Langmuir and Freundlich isotherm parameters for the adsorption of Cr(vi)

	Parameters	SG-APTES	SG-AEMH	MS-APTES	MS-PEI
Langmuir	*q*_max_ (mg/g)	10.34	63.29	34.09	50.26
		±2.26	±2.12	±1.15	±1.11
	*b* (L mg^−1^)	0.15	0.31	0.56	0.60
		±0.05	±0.01	±0.01	±0.02
	*R*_L_	0.24	0.13	0.57	0.07
		±0.03	±0.02	±0.02	±0.01
	*R* ^2^	0.88	0.99	0.94	0.97
Freundlich	*K*_F_ (mg g^−1^)	2.61	20.92	2.80	22.67
		1.26	±1.50	±1.52	±1.31
	1/*n*	0.32	0.30	0.54	0.23
		±0.15	±0.01	±0.01	±0.01
	*R* ^2^	0.31	0.87	0.86	0.98

In case of MS-PEI, the value of *R*^2^ for the Freundlich isotherm model (0.98) was slightly higher than that for the Langmuir (0.97). Furthermore, the higher values of *b* (Langmuir constant) for SG-AEMA (0.31 L mg^−1^) and MS-PEI (0.60 L mg^−1^) indicated a stronger attraction of Cr(vi) ions on the polymer functionalized surfaces compared to the monolayer based adsorbent surfaces. The maximum adsorption capacities (*q*_max_) for SG-AEMH (63.29) and MS-PEI (50.26) are higher than SG-APTES (10.34) and MS-APTES (34.09). The calculated values of 1/*n* range between 0 and 1 for all adsorbents imply that adsorption process was chemical in nature. The values of 1/*n* depict adsorption process is more heterogeneous for MS-PEI (0.23) than for SG-AEMH (0.30). Moreover, the calculated value of *R*_L_ is also in the required range of 0 < *R*_L_ < 1 for SG-AEMH (0.13) and MS-PEI (0.07), signifying a favourable adsorption of Cr(vi).^49,69–71^

### Adsorption kinetics

Adsorption kinetic is one of the most important parameter, which represents the adsorption efficiency. It determines the adsorbate uptake rate and evaluates the equilibrium time required for the sorption isotherm. To understand the kinetic mechanism of the adsorption process, pseudo-first-order^72^ and pseudo-second-order^73^ kinetics models were applied to fit the kinetic data. The linear form of pseudo-first-order kinetic equation is expressed by following equation:5
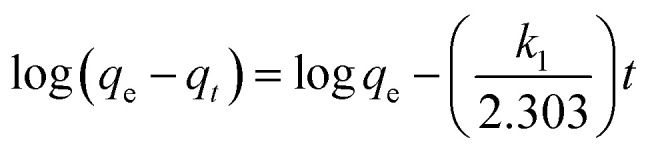
where *q*_e_ and *q*_*t*_ are the amount of metal ions adsorbed on the adsorbent in mg g^−1^ at equilibrium and at time *t*, respectively, and *k*_1_ is the constant of first-order adsorption (min^−1^).

The pseudo-second-order kinetic rate equation is linearly expressed as following:6
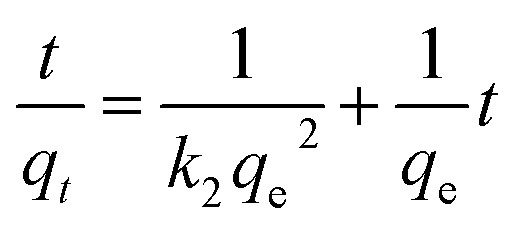
where *k*_2_ is the pseudo-second-order rate constant at the equilibrium (g mg^−1^ min^−1^) that can be determined experimentally. The kinetics parameters and correlation coefficients were calculated from the linear plots and are listed in Table 3. The adsorption data of SG-APTES, MS-APTES, SG-AEMH and MS-PEI fit the pseudo-second-order model with higher correlation coefficient (*R*^2^) values. The theoretical *q*_e_ values for the adsorbents were very close to the experimental *q*_e_ values in the case of second-order kinetics. These results suggest that the rate limiting step involves chemisorption of the adsorbate onto the adsorbent.^74–77^

**Table 3:** Kinetic parameters for the adsorption of Cr(vi) ions

	Parameters	SG-APTES	SG-AEMH	MS-APTES	MS-PEI
Pseudo-first-order	*q*_e_ (mg g^−1^) calculated	4.77	4.45	5.95	1.71
		±0.13	±0.22	±0.61	±0.06
	*q*_e_ (mg g^−1^) experimental	8.43	36.38	8.98	19.41
		±0.03	±0.03	±0.02	±0.02
	*k*_1_ (min^−1^)	0.05	0.10	0.08	0.06
		±0.03	±0.03	±0.01	±0.01
	*R* ^2^	0.92	0.04	0.57	0.30
Pseudo-second-order	*q*_e_ (mg g^−1^) calculated	10.08	37.74	10.87	19.89
		±0.11	±0.26	±0.2	±0.08
	*q*_e_ (mg g^−1^) experimental	8.43	36.38	8.98	19.41
		±0.03	±0.03	±0.02	±0.02
	*k*_2_ (g mg^−1^ min^−1^)	0.12	0.27	0.12	0.27
		±0.02	±0.02	±0.02	±0.02
	*R* ^2^	0.98	0.99	0.97	0.99

### Adsorption thermodynamics

To evaluate the thermodynamic feasibility and spontaneous nature of the adsorption process, thermodynamic parameters including the entropy (Δ*S*°), enthalpy (Δ*H*°) and standard Gibbs free energy (Δ*G*°) were calculated.^78–80^

The magnitude of Δ*G*° was calculated from the following equation:

7 Δ*G*° = −*RT* ln *K*

Where *K* is the equilibrium constant, *T* is the absolute temperature (K), and *R* is the universal gas constant (8.314 J mol^−1^ K^−1^).

The change in enthalpy Δ*H*° and Δ*S*° can be determined from the following equation:8
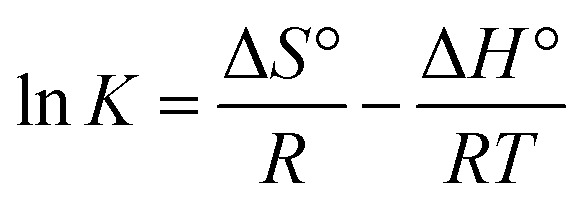


The equilibrium constant *K* can be calculated as expressed in eqn (9):9
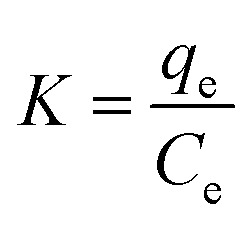
where, *K* is the equilibrium constant, *q*_e_ is the solid phase concentration at equilibrium (mg L^−1^) and *C*_e_ is the equilibrium concentration in solution (mg L^−1^).

The values of the thermodynamic parameters are given in Table 4. The negative values of Δ*G*° implied that the adsorption process was feasible and spontaneous.

**Table 4:** Thermodynamic parameters for the adsorption of Cr(vi) ions

Parameters	SG-APTES	SG-AEMH	Parameters	MS-APTES	MS-PEI
Δ*G*° (kJ mol^−1^)	−3.50	−2.22	Δ*G*° (kJ mol^−1^)	−0.66	−3.11
	±1.31	±1.53		±0.33	±0.50
Δ*H*° (kJ mol^−1^)	−6.69	−7.13	Δ*H*° (kJ mol^−1^)	−1.59	−16.45
	±1.60	±1.30		±0.70	±0.58
Δ*S*° (kJ mol^−1^ K)	−0.049	0.021	Δ*S*° (kJ mol^−1^ K)	−0.010	0.012
	±0.022	±0.020		±0.01	±0.02
*K*	0.24	11.64	*K*	0.77	33.44
	±0.09	±0.13		±0.01	±0.10

In addition, the negative values of Δ*H*° suggested that the adsorption of Cr(vi) onto SG-AEMH, SG-APTES, MS-PEI and MS-APTES was exothermic in nature.

The positive values of Δ*S*° for SG-AEMH and MS-PEI exhibited the increasing randomness at the solid–liquid interfaces during the adsorption of metal ions on the adsorbents and could be due to some structural changes in the adsorbents. While, the negative values of Δ*S*° for SG-APTES and MS-APTES suggested that the randomness decreased at the solid/solution interface as a results of Cr(vi) adsorption onto the surface of adsorbents. This implied that the adsorption process was energetically stable.^81–84^

The molar entropy of adsorption is

10 Δ_ad_*S*_*m*_ = *S*_*m*_^*σ*^ − *S*_*m*_^l^

where ad is adsorption, *m* is molar, *σ* is interface, and l is solution phase (liquid).

While *S*_*m*_^*σ*^ can be calculated from following equation:11
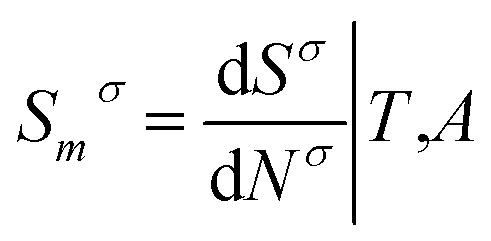
where *N*^*σ*^ is moles of adsorbate at the interface, *T* is temperature and *A* is the total area of the adsorbent.

For monolayer based surfaces (SG-APTES and MS-APTES), Δ*S*_*m*_ is negative (the entropy of adsorbates at the interface is smaller than the entropy in the solution). Therefore, entropically driven adsorption is restricted. This is because, the entropy of molecules on the monolayer coated surface is much lower than in solution phase since vibrational, rotational and also translational degrees of freedom are restricted at the interface. However, the polymer decorated silica gel (SG-AEMH) and mesoporous silica (MS-PEI) showed positive entropy change upon adsorption, since molecules have more freedom to move compared to monolayer. Besides, the positive value of entropy also means that the change of amount of adsorbate as a function of entropy at the interface is larger than in the solution. Therefore, entropy driven adsorption is more favorable for polymer functionalized solid adsorbents as compared to their monolayer counterparts.

### Desorption

**Fig. 9:**
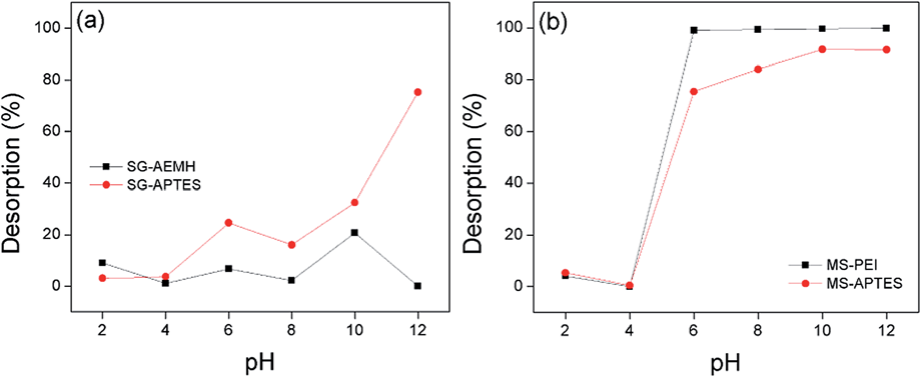
Effect of pH on desorption from (a) SG-APTES and SG-AEMH (b) MS-APTES and MS-PEI.

A successful desorption process must restore the adsorbent close to its initial properties for effective reutilization. Sorbent regeneration is significant in evaluating the competitiveness of the adsorbent system. The regeneration of adsorbents was monitored by different eluting agents (NaOH, NaNO_3_, mixture of NaOH with NaNO_3_ (1 : 1)). It was observed that best desorption results for SG-AEMH (up to 70%) were obtained by using NaNO_3_ and for SG-APTES (up to 74%) were obtained by using NaOH, while for MS-PEI (up to 90%) and MS-APTES (up to 88%) the best desorption results were obtained by using NaOH. The effect of pH on desorption was also explored. The maximum desorption was observed at basic conditions, due to an increase in the negative species in the media (Fig. 9).

**Fig. 10:**
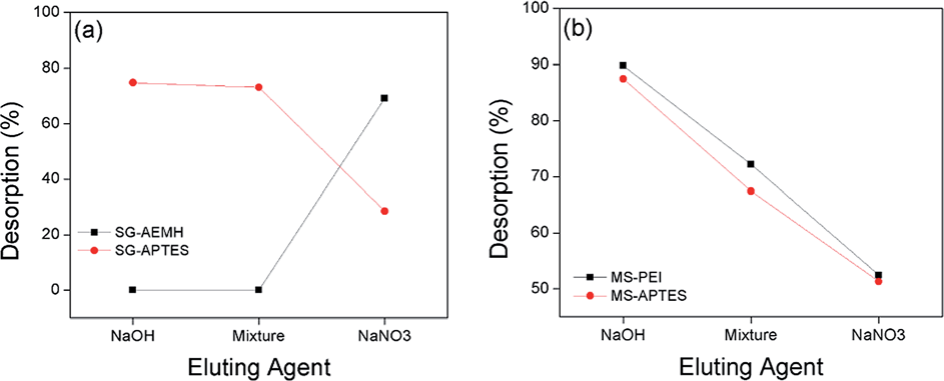
Effect of eluting agents on desorption from (a) SG-APTES and SG-AEMH (b) MS-APTES and MS-PEI.

At higher pH (pH = 10), desorption was up to 20% for SG-AEMH, whereas desorption percentage was up to 75% in the case of SG-APTES at pH = 12, while, desorption was up to 98% for MS-PEI and up to 91% in the case of MS-APTES at pH = 12 (Fig. 10).^85,86^

### Regeneration/reusability

**Fig. 11:**
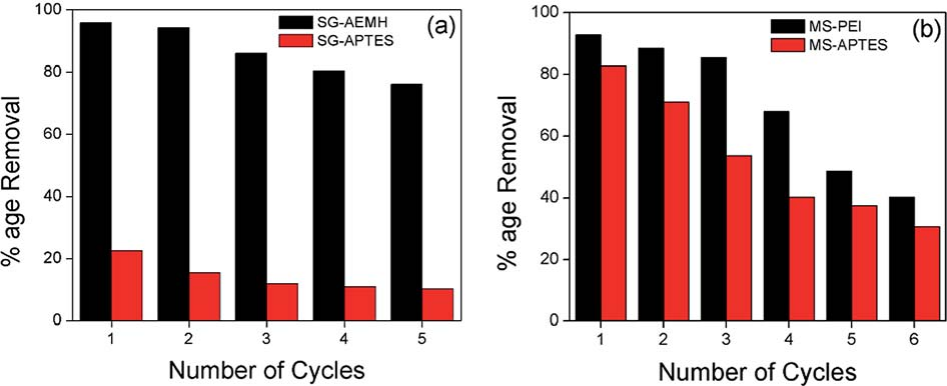
Reusability of (a) SG-APTES and SG-AEMH, (b) MS-APTES and MS-PEI.

The regeneration ability of the adsorbent reduces the process cost and assesses the competence of adsorption systems. To investigate the reusability, Cr(vi) loaded adsorbents were washed with 0.1 M NaOH solution and then rinse with deionized water to neutrality and reconditioned for reuse. The results showed that a performance drop of 21% and 57% was observed in the adsorption capacity of SG-AEMH and SG-APTES between the 1st and 5th cycles, respectively. MS-PEI could be effectively reused up to sixth adsorption–desorption cycles with 56% performance loss while, a drop of 63% was observed in the adsorption capacity of MS-APTES up to sixth cycle (Fig. 11).^87,88^

## Conclusions

In summary, silica gel was functionalized with polymer to improve the adsorption behaviour towards Cr(vi). The removal efficiency of polymer functionalized silica (SG-AEMH) was compared with the mesoporous silica tethered with a branched polymer (MS-PEI). The polymer decorated silica gel (SG-AEMH) and mesoporous silica (MS-PEI) exhibited better adsorption capacities as compared to the monolayer based SG-APTES and MS-APTES platforms. The prepared silica sorbents exhibited attractive characteristics, such as high adsorption capacity, fast adsorption kinetics, and superior regeneration performance. The adsorption process of SG-AEMH was well described with a Langmuir model while Freundlich model gave a good fit for the adsorption data of MS-PEI. Pseudo-second order equation gave a better correlation for the adsorption data of SG-AEMH and MS-PEI. The thermodynamic study indicated that the adsorption processes were spontaneous and exothermic for SG-AEMH and MS-PEI based sorbents. The present study revealed that SG-AEMH and MS-PEI are promising materials for the removal of Cr(vi) ions from aqueous media and could be regenerated and reused up to five cycles for SG-AEMH and six cycles for MS-PEI that highlight their economic viability.

## Conflicts of interest

There are no conflicts to declare.

## Supplementary Material
